# Point-of-care manufacturing: a single university hospital’s initial experience

**DOI:** 10.1186/s41205-021-00101-z

**Published:** 2021-04-22

**Authors:** Jose Antonio Calvo-Haro, Javier Pascau, José Manuel Asencio-Pascual, Felipe Calvo-Manuel, Maria José Cancho-Gil, Juan Francisco Del Cañizo López, María Fanjul-Gómez, Roberto García-Leal, Guillermo González-Casaurrán, Manuel González-Leyte, Juan Antonio León-Luis, Lydia Mediavilla-Santos, Santiago Ochandiano-Caicoya, Ramón Pérez-Caballero, Almudena Ribed-Sánchez, Javier Río-Gómez, Eduardo Sánchez-Pérez, Javier Serrano-Andreu, Manuel Tousidonis-Rial, Javier Vaquero-Martín, Sonia García San José, Rubén Perez-Mañanes

**Affiliations:** 1 Advanced Planning and 3D Manufacturing Unit, Hospital General Universitario Gregorio Marañón, Calle Doctor Esquerdo, 46, 28007 Madrid, Spain; 2 Faculty of Medicine, Universidad Complutense, Madrid, Spain; 3 Instituto de Investigación Sanitaria Gregorio Marañón, Madrid, Spain; 4 Departamento de Bioingeniería e Ingeniería Aeroespacial, Universidad Carlos III de Madrid, Madrid, Spain; 5 Clínica Universidad de Navarra, Madrid, Spain

**Keywords:** 3D printing, Manufacturing university hospital, POC manufacturing, Preoperative planning, Biomodel, Surgical guides, Custom implants

## Abstract

**Background:**

The integration of 3D printing technology in hospitals is evolving toward production models such as point-of-care manufacturing. This study aims to present the results of the integration of 3D printing technology in a manufacturing university hospital.

**Methods:**

Observational, descriptive, retrospective, and monocentric study of 907 instances of 3D printing from November 2015 to March 2020. Variables such as product type, utility, time, or manufacturing materials were analyzed.

**Results:**

Orthopedic Surgery and Traumatology, Oral and Maxillofacial Surgery, and Gynecology and Obstetrics are the medical specialties that have manufactured the largest number of processes. Working and printing time, as well as the amount of printing material, is different for different types of products and input data. The most common printing material was polylactic acid, although biocompatible resin was introduced to produce surgical guides. In addition, the hospital has worked on the co-design of custom-made implants with manufacturing companies and has also participated in tissue bio-printing projects.

**Conclusions:**

The integration of 3D printing in a university hospital allows identifying the conceptual evolution to “point-of-care manufacturing.”

## Background

3D printing is a disruptive technology and represents a paradigm shift in healthcare delivery [1]. The term “3D Printing” refers to the transformation of a digital model into a tangible three-dimensional object, with examples including anatomical models (biomodels) [2, 3], medical devices [4], surgical instruments [5, 6], custom-made implants [7–10], drugs [11–13], or even organs and tissues [14–16]. 3D printing enables patient-specific precision medicine with improved medical communication, and reports in the literature present shorter medical procedures, personalized medical devices, or simulation for medical training [17, 18].

Greater accessibility to 3D printing software and hardware technologies has enabled hospital-based 3D printing, showing how lead times and costs can be reduced [19]. Hospital 3D printing labs generate value in personalized medicine by assembling a professional team and the necessary resources [20–22]. With the proper quality and safety infrastructure, hospital-based 3D printed products can be affordable and incorporate a hub-and-spoke model, where more complex manufacturing university hospitals are identified as “hubs,” and smaller, less diverse labs serve as the “spokes.” This approach enables each institution to adjust resources accordingly, objectively identifying each partners’ strengths and limitations. The result is the creation of strong 3D printing alliances [20, 23].

3D printing within a hospital is a paradigm shift, moving away from traditional industry-based manufacturing limitations to propose custom implants [24–27] or bioprinting [28–30]. These hospital-based labs have been referred to as point-of-care (POC) manufacturing and require pecuniary responsibility, technical competence, updated physical resources, and engaged human resources [20].

Hospital General Universitario Gregorio Marañón has been developing the POC 3D-printing model since 2015. This study aims to present the experience from this integration of 3D printing technology in our hospital. Our results show how POC manufacturing workflow is a paradigm of personalized medicine in all steps, from clinical indication and surgical planning to the design and manufacturing of patient-specific solutions.

## Methods

We present an observational, descriptive, retrospective, and monocentric study of 3D printing experience in the Advanced Planning and Manufacturing 3D Unit (UPAM3D) at Hospital General Universitario Gregorio Marañón (Madrid, Spain). We include all production recorded from the first 3D-printed case in November 2015 to March 2020, when the activity at UPAM3D was temporarily interrupted due to the COVID-19 pandemic.

3D printing transforms a digital model into a real and tangible three-dimensional product. Digital 3D models can be obtained from different sources: digital radiological studies, three-dimensional scanning, computational design (CAD), or reverse engineering. The products are built layer by layer from the digital model using different technologies and materials depending on the final application [31, 32].

In this study, we have defined the following variables to analyze the 3D printing activity at UPAM3D:

- Required product: Describes the type of product that is requested by the original user. It can be an anatomical model, a surgical guide/instrument for an interventional procedure, or a surgical navigation tool.
- Product utility: It defines the primary function of the product. Although these products have great value as a communication tool in most cases, it has been described as such only when communication was the essential value of the product. Other alternatives are teaching, instrumental, research, preoperative planning, intraoperative utility, and others.
- Input data: Describes the original data provided by the requesting user. Possible values are DICOM files [DICOM], 3D digital model design [3D model], or other materials. This last case could be a drawing or an existing tool with design improvement suggestions, which would be reverse-engineered to obtain a 3D model.
- Work time: Time allocated by the medical and technical team in all steps required to complete the project and obtain a final delivery product. This included 3D model design, 3D printing preparation (slicing), and post-processing of the 3D-printed result after manufacturing.
- 3D printing time: Time required by the 3D printer to manufacture the product.
- Quantity of 3D printing material: Weight of the material (in grams) used by the printer for each product.
- Type of 3D printing material: Type of material used to manufacture the product that depends on the 3D printing technology. Two types of technology were available during this study: fused deposition modeling (FDM), which prints fusing different compositions of plastic materials, and vat photopolymerization (SLA technology), a process in which a liquid photopolymer (resin) is converted into solid material when illuminated by a laser.

Quantitative variables are described as mean and median values, while qualitative variables are presented as numbers and percentages.

## Results

**Fig. 1:**
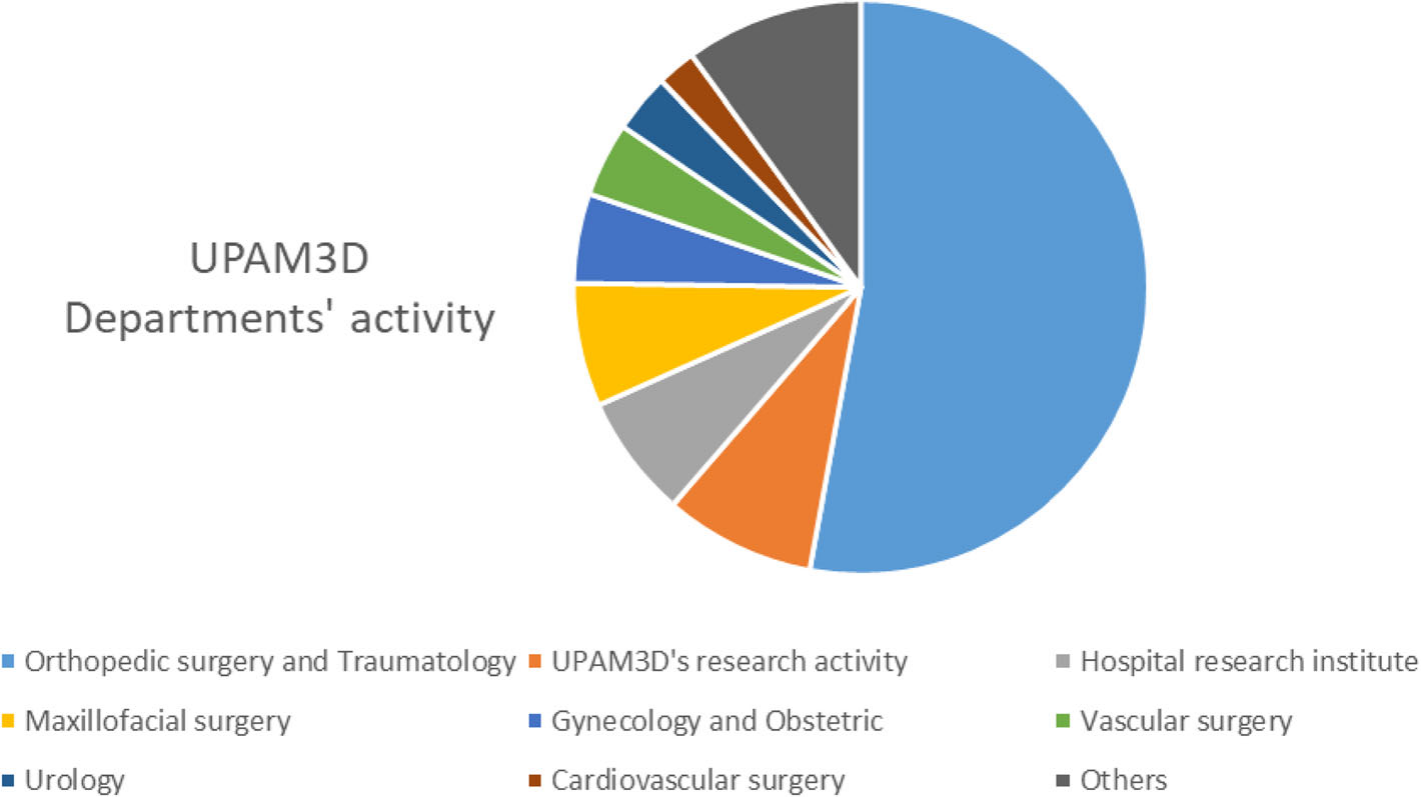
Distribution of the UPAM3D activity for departments that have requested product manufacturing

Figure 1 summarizes the activity of the different departments for a total of 907 models 3D printed during the study. The detailed data is shown in Table 2. Most of them were clinical departments, being the most active Orthopedic Surgery and Traumatology, Maxillofacial Surgery, and Gynecology and Obstetrics with 52.81%, 6.93%, and 5.06% of the processes, respectively. Apart from the clinical activity, the Hospital Research Institute, and UPAM3D demanded several research products.

**Fig. 2:**
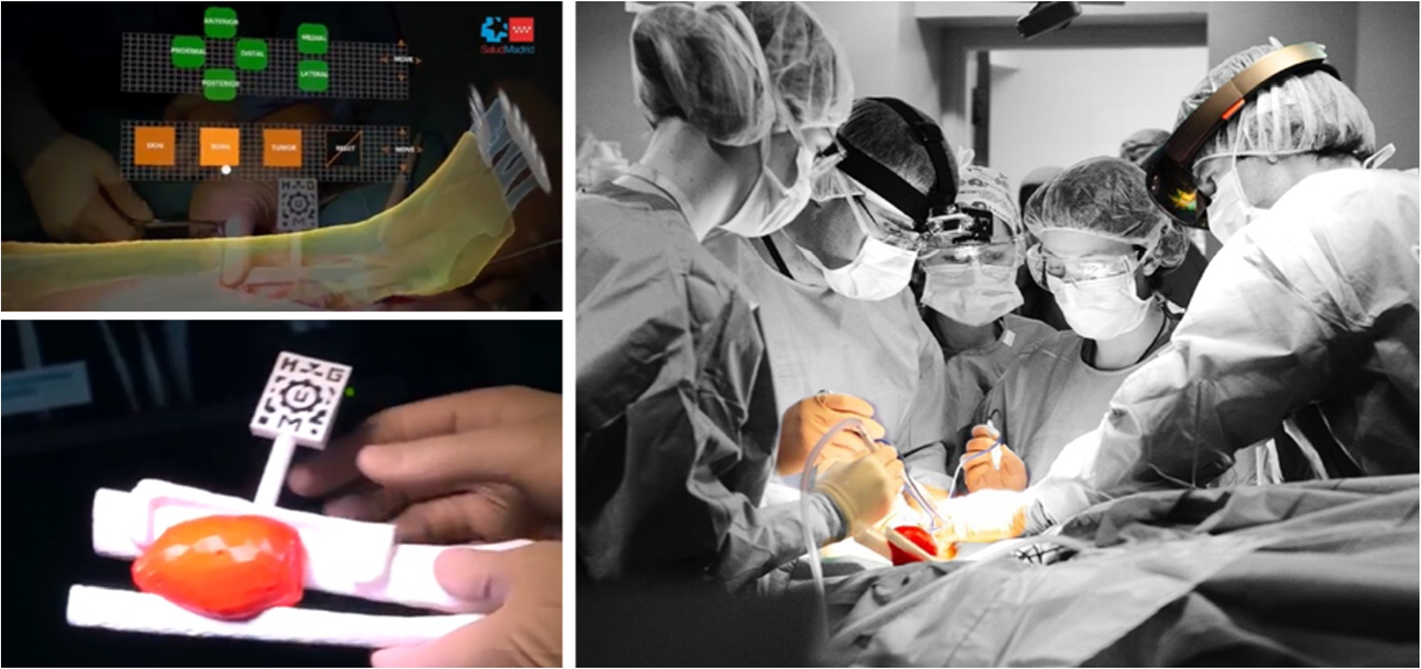
Surgical procedure: combining augmented reality and 3D printing. Ewing tumor. Location, right leg. Date, 2018

The products requested were anatomical models in 87.02% of cases and surgical guides in 11.66%. The remaining cases (1.32%) were patient-specific instruments for surgical procedures in which 3D printing was integrated with navigation or augmented reality, providing added value, as in the example displayed in Fig. 2 [33, 34].

The utility has been different depending on the type of product manufactured. Anatomical models were primarily manufactured for preoperative planning (38.61%), communication (9.35%), or research (18.26%). On the other side, surgical guides or instruments have mainly been used intraoperatively (Table 1).

**Table 1:** Distribution of required products for each product utility

	Product utility							
	Communication	Teaching	Instrumental	Research	Intraoperative utility	Preoperative planning	Others	Total (%)
**Required product**								
**Surgical guide/interventional procedure**	0.11			0.11	11.44			11.66
**Anatomical model**	9.35	12.54	0.55	18.26	0.66	38.61	7.04	87.02
**Navigation tool**				0.77	0.44	0.11		1.32
**Total (%)**	**9.46**	**12.54**	**0.55**	**19.14**	**12.54**	**38.72**	**7.04**	**100.00**

When we analyze the type of products requested by each department (Table 2), we can observe that anatomical models are the most common. However, the profile for some surgical departments is different. For instance, Pediatric Surgery requested positioning guides in 70% of the cases.

**Table 2:** Distribution of products and input data by departments

	Surgical guide/interventional procedure				Anatomical model				Navigation			Total (%)
	3D model	DICOM	Other	Total	3D model	DICOM	Other	Total	3D model	DICOM	Total	
**Anesthesiology**					0.99		0.22	1.21				1.21
**Vascular surgery**	0.11	0.11		0.22	1.32	2.53	0.11	3.96				4.18
**Biochemistry**					0.44			0.44				0.44
**Cardiology**					0.44	0.11		0.55				0.55
**Cardiovascular surgery**					1.43	0.11	0.66	2.20				2.20
**General surgery**					0.44	0.11	0.11	0.66		0.11	0.11	0.77
**Maxillofacial surgery**	0.44	1.43		1.87	1.43	3.19	0.44	5.06				6.93
**Orthopedic surgery and traumatology**	4.95	1.98	0.44	7.37	13.97	28.93	2.09	44.99	0.33	0.11	0.44	52.81
**Pediatric surgery**	0.11	0.66		0.77		0.33		0.33				1.10
**Plastic surgery**					0.22	0.33	0.11	0.66				0.66
**Thoracic surgery**					0.22	1.21		1.43				1.43
**Hospital pharmacy**							0.22	0.22				0.22
**Gynecology and obstetric**					4.29	0.77		5.06				5.06
**Hospital research institute**					4.07	0.11	1.98	6.16	0.77		0.77	6.93
**Physical medicine and rehabilitation**					0.11	0.11		0.22				0.22
**Neonatology**						0.22		0.22				0.22
**Neurosurgery**						1.43		1.43				1.43
**Radiotherapy oncology**					0.22	0.11		0.33				0.33
**Otorhinolaryngology**					0.11			0.11				0.11
**Radiology**					0.33	0.55	0.11	0.99				0.99
**Radiophysic**							0.11	0.11				0.11
**UPAM3D’s research activity**					6.16	0.55	1.76	8.47				8.47
**Urology**		1.43		1.43	0.66	1.21		1.87				3.30
**Other departments**					0.11		0.22	0.33				0.33
**Total (%)**	**5.61**	**5.61**	**0.44**	**11.66**	**36.96**	**41.91**	**8.14**	**87.02**	**1.10**	**0.22**	**1.32**	**100.00**

**Fig. 3:**
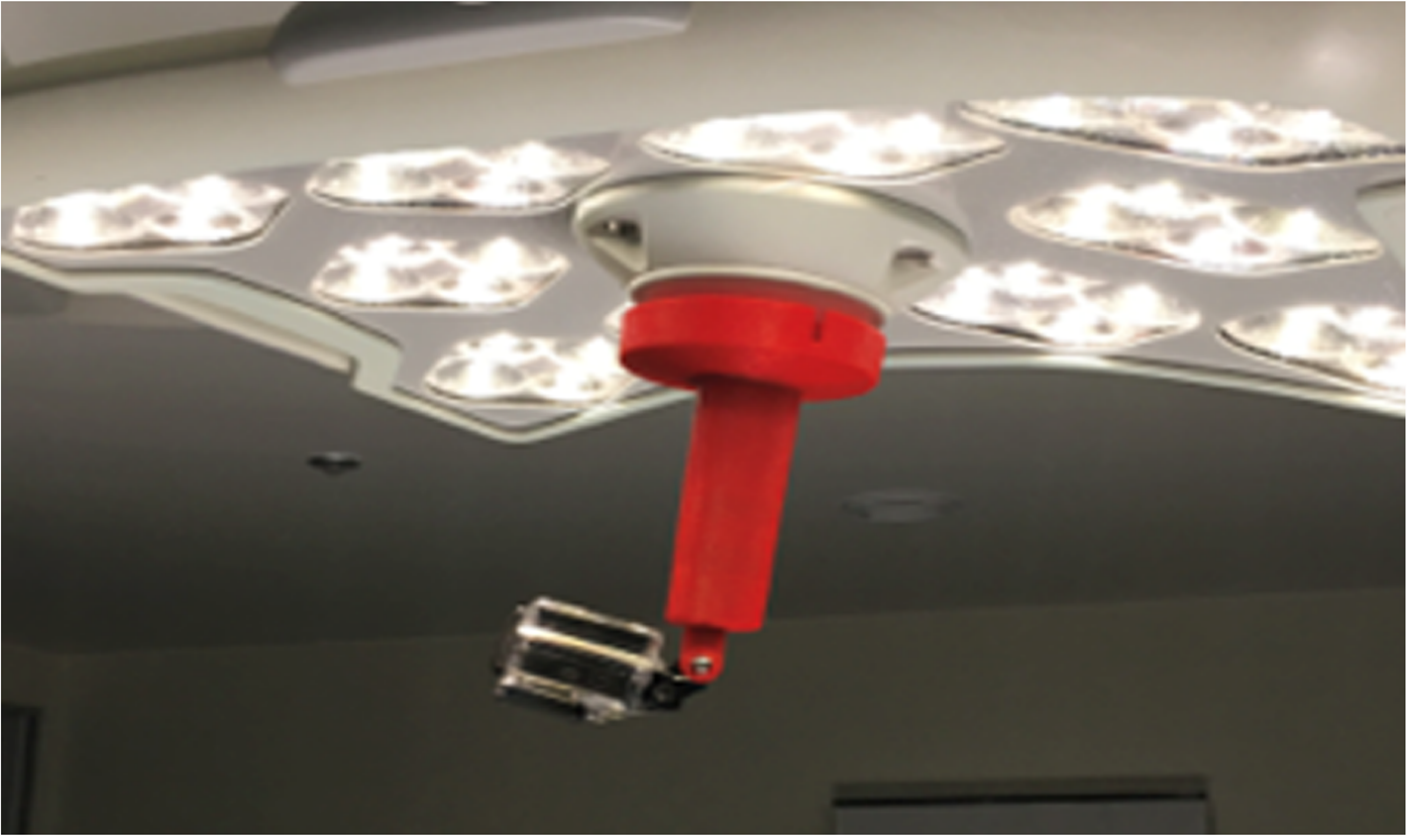
Reverse engineering. Sterilizable surgical light handle with camera attachment. Date, 2016

Table 2 details the distribution of input data provided to perform the 3D printing process, with DICOM images, 3D models, and other materials in 47.74%, 43.67%, and 8.58% of the cases. Figure 3 shows an example of a 3D-printed camera attachment for a surgical lamp belonging to this last category. When analyzing each department’s input data, we can observe that the Neurosurgery provided DICOM images in all their requests. At the same time, other departments were able to prepare a 3D model for their products. Orthopedic Surgery and Traumatology is a good example: their input data was a 3D model in 31% and 67.16% of their anatomical models and surgical guides, respectively.

Table 3 shows the technical details of the 3D printing projects during the study period. The total working hours were 8896, with an average of 9.79 h (median 3 h) per process. The total running time for all 3D printers was 10,271 h, with an average of 11.29 h (median 6 h) per process. However, working time and 3D printing time were different depending on the type of product and the input data. The production time for a product must consider both the working time of the staff involved in the design and printing processes (medical and technical team) and the manufacturing time required by the 3D printer.

**Table 3:** Technical details of the 3D printing projects

	Surgical guide/interventional procedure				Anatomical model				Navigation			Total
	3D model	DICOM	Other	Total	3D model	DICOM	Other	Total	3D model	DICOM	Total	
**Work time (hours)**												
Mean	13.39	3.21	6.75	8.24	1.85	5.73	10.59	10.07	1	30.5	5.91	9.79
Median	10	2	1	4	3	2	3.5	2	10	1.5	6	3
**3D printing time (hours)**												
Mean	2.96	7.78	14.75	5.72	8.07	16.78	6.23	12.08	4.9	25	8.25	11.29
Median	2	4	6.5	2	4.5	12	2	7	7	2	5	6
**Quantity 3D printing material (grams)**												
Mean	41.74	74.96	193	63.41	90.64	149.53	73.4	117.31	63.6	157.5	79.25	110.65
Median	30	72	61	42.5	51	97	29.5	66	83.5	25.5	81	64

**Fig. 4:**
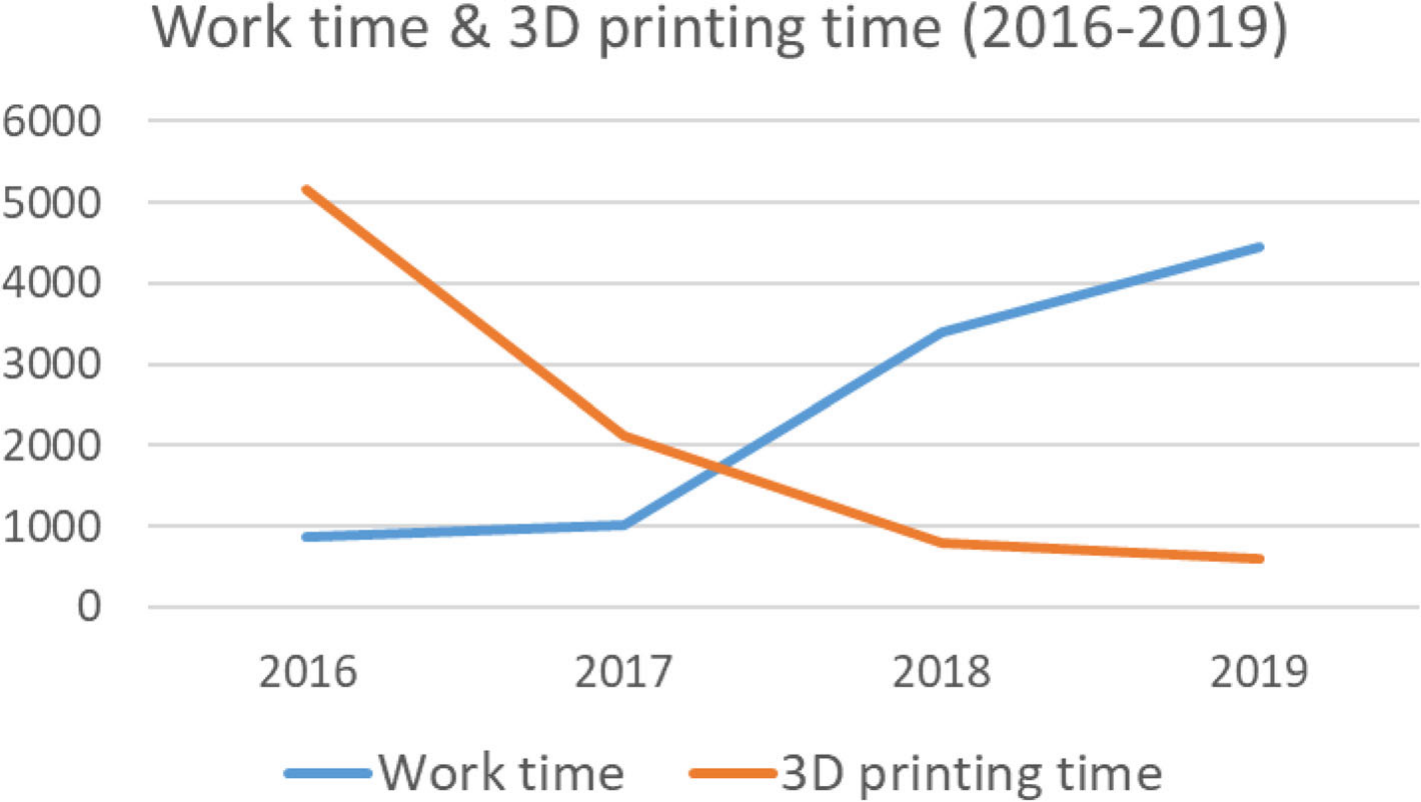
Working and 3D printing times. Annual distribution. Data for 2015 and 2020 are not included (incomplete)

The annual distribution of working and printing hours (Fig. 4) shows two lines that cross during the study period, due to a more significant number of working hours in the first years with increased printing times in recent years. This behavior can be explained by the increase in the complexity of the products being printed, combined with a reduction of the segmentation and image processing times required to obtain virtual 3D models from input data.

**Fig. 5:**
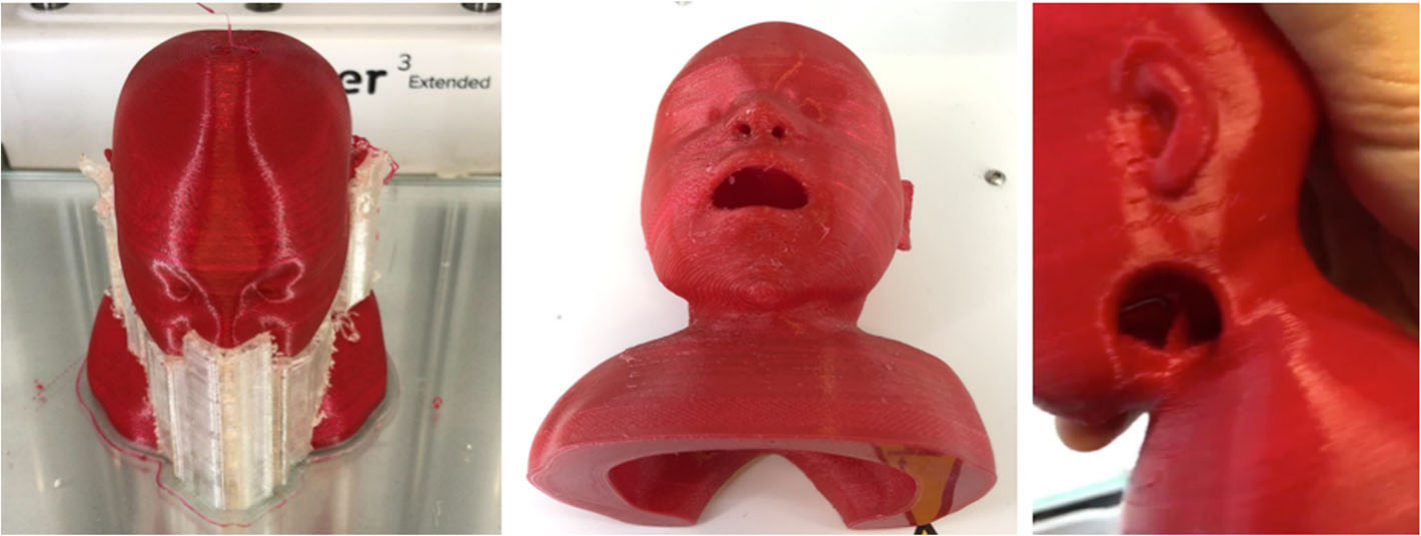
3D printing materials (TPU, PVA). Pediatric intubation training. Date, 2018

**Fig. 6:**
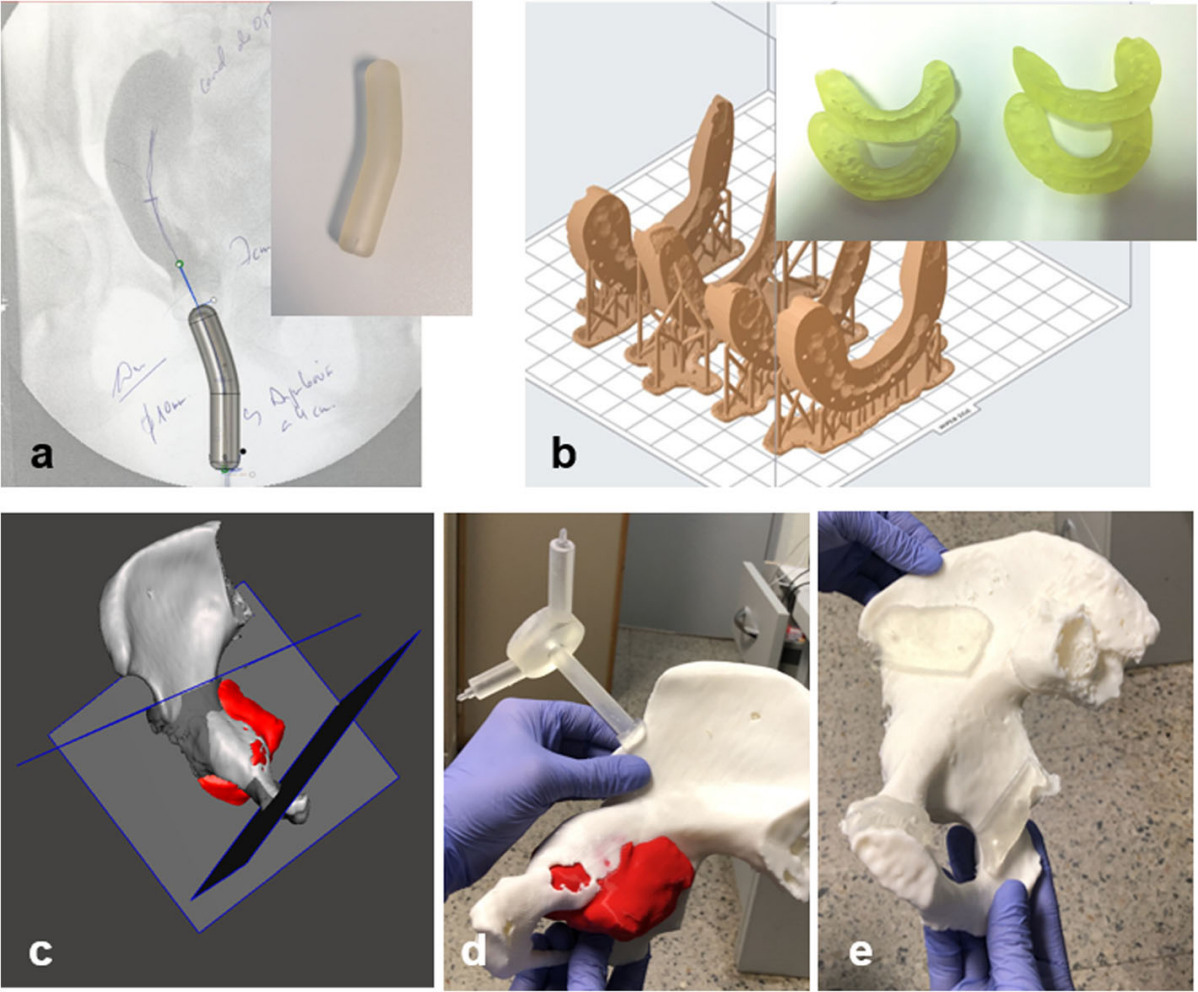
3D printing materials (biocompatible resin). Surgical guides. Clinical applications: urogenital malformation surgery (**a**), orthognathic surgery (**b**), orthopedic oncology (**c**-**e**)

Total 3D printing material consumed was 100,587 g, with an average of 110.65 g (median of 64 g) per process (Table 3). 3D printers with FDM technology have been the most used, accounting for 94.65% of 3D printing material, being polylactic acid (PLA) the most widely used (79.30%). This is a rigid material replicating bone structures with remarkable realism. Many other materials have been tested in specific projects, some of them flexible such as thermoplastic polyurethane (TPU) or Filaflex (thermoplastic elastomer based on polyurethane and certain additives). They allow replicating vascular structures, solid organs, or muscular-tendon structures. Supporting materials, such as polyvinyl alcohol (PVA), facilitate post-processing, and improve printing quality. The contribution of PVA is depicted in Fig. 5, showing a phantom with internal cavities that could be manufactured with rigid supports. The recent introduction of SLA resins, biocompatible, and certified for medical use, allowed us to respond to an increased demand for positioning guides or patient-specific instruments. In total, 4.95% of products have been printed with biocompatible resins (Dental SG Resin and Dental LT Clear Resin, Formlabs), representing 5.35% of the consumed material. Figure 6 shows several projects printed in biocompatible SLA resins.

**Fig. 7:**
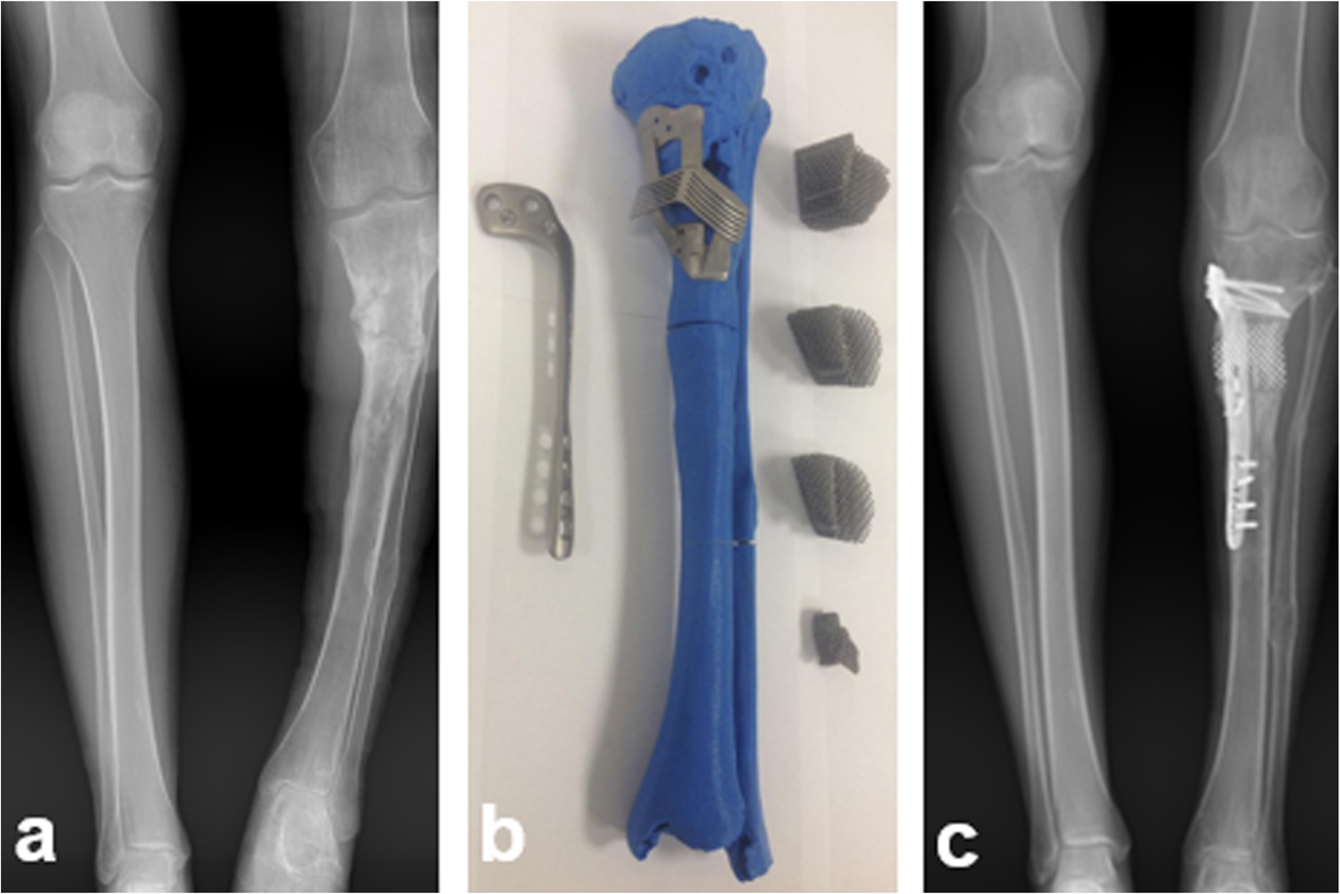
Customized implant. Preoperative X-ray of a tibial deformity (**a**). Anatomical model and customized implants (**b**). Post-operative X-ray (**c**) Date, 2017

**Fig. 8:**
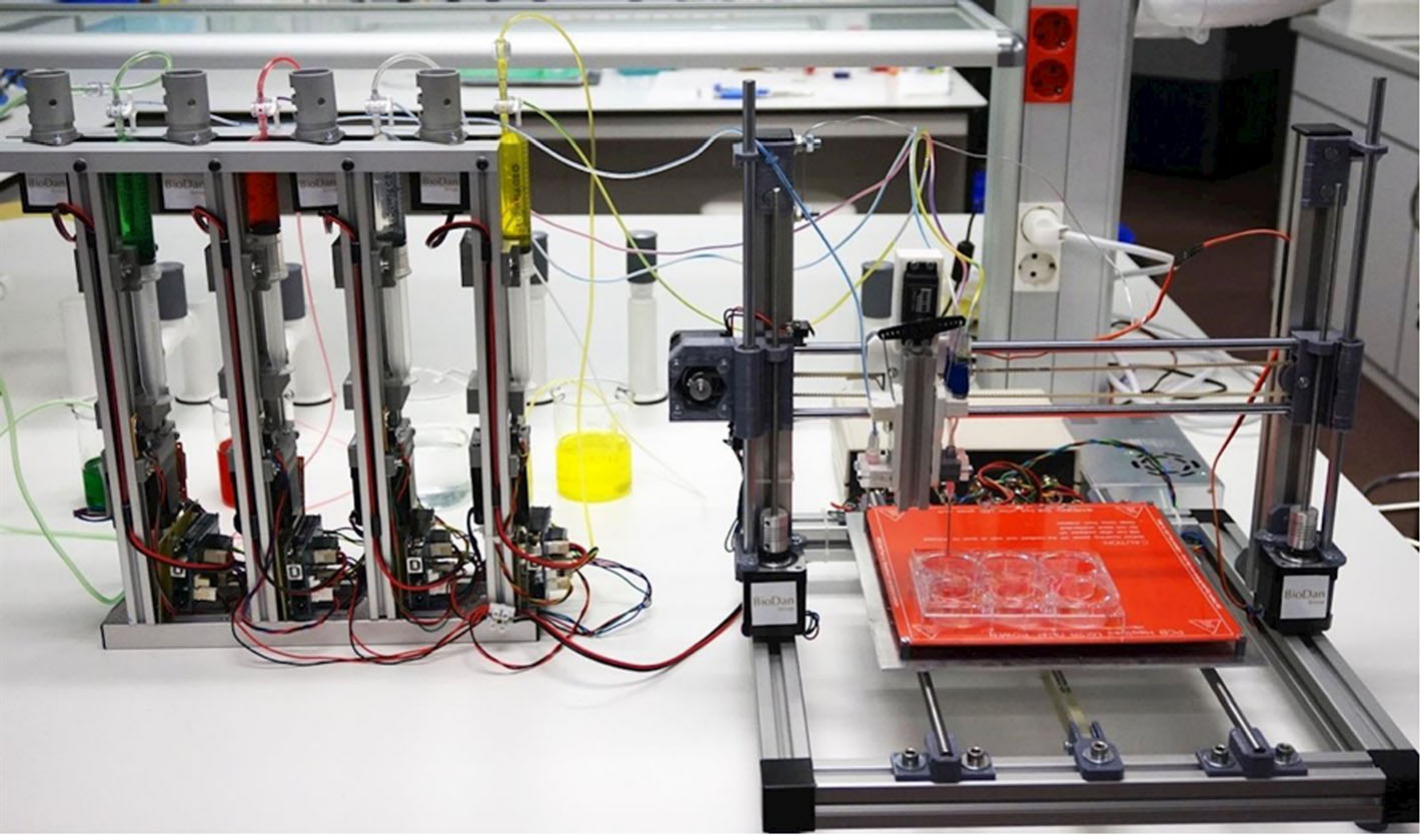
Human skin bioprinting. Date, 2016

Our experience as a manufacturing university hospital has increased our capacity to work with different commercial companies in the sector, participating in the co-design of personalized implants [35] (Fig. 7) or collaborating with external research groups in bioprinting [36, 37] (Fig. 8).

## Discussion

**Fig. 9:**
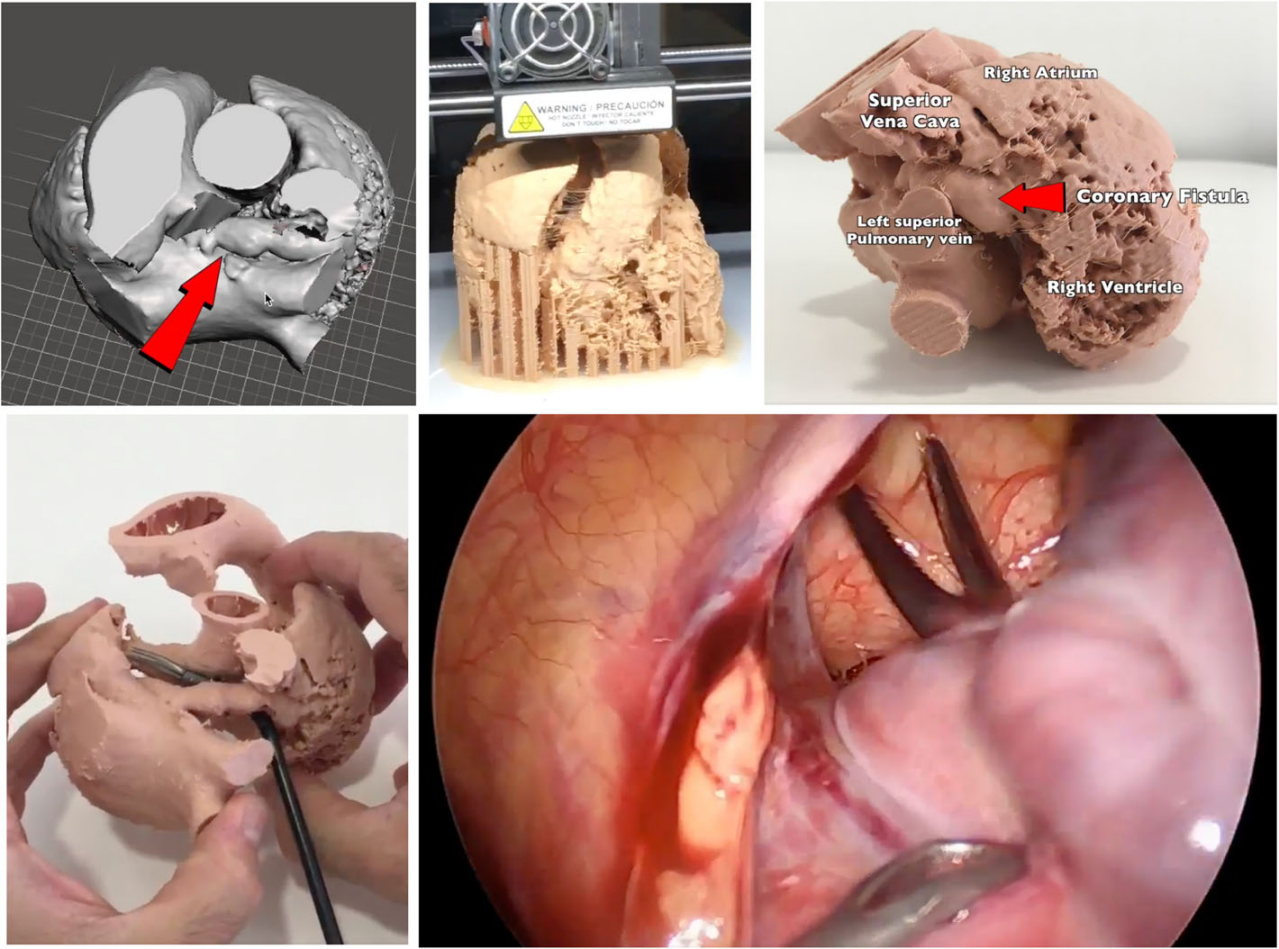
Preoperative planning. Congenital heart defect. Date, 2017

3D printing addresses the growing complexities in healthcare and enables a more sustainable future as a scalable and cost-effective technology. It is a patient-specific process (Fig. 9) that increases the efficiency in different steps of the treatment workflow, bringing higher level of customization and predictability, as proposed by the GIRFT methodology (Getting It Right First Time) [19, 38–42].

Hospital General Universitario Gregorio Marañón has pioneered the transversal implementation of hospital 3D printing with an “in-house” medical 3D printing laboratory integrated into the clinical workflow of more than 20 medical-surgical specialties. The hospital has obtained authorization from the local government to manufacture medical devices, in compliance with the international standard ISO 13485 for quality management systems for medical products [43–48]. During the first 2 years, UPAM3D activity focused on identifying and optimizing specific software and hardware, materials, and manufacturing parameters. This profile is reflected in the results, showing higher involvement of the research departments and a large number of working hours. The expertise acquired during this initial phase resulted in a decrease in working hours every year, and the inclusion of new requesting users from clinical departments.

It is important to identify the clinical areas gaining the most benefits from integrating 3D printing in their clinical workflow. The Radiological Society of North America 3D printing group (3D Special Interest Group RSNA) states that musculoskeletal, craniofacial, vascular, or congenital heart defects are the specialties in which it is most efficient to use anatomical models [49]. Our results show that Orthopedic Surgery and Traumatology, Maxillofacial Surgery, Gynecology and Obstetrics, and Vascular Surgery accounted for 68.98% of the total activity. Research activity (UPAM3D or the Research Institute) reported 15.4%, and the remaining 15.62% (142 cases) included other clinical departments. Our role as a manufacturing university hospital has allowed adaptation and optimization of response times. This is important in surgical specialties such as Traumatology [50] or Vascular Surgery [51], where traditional manufacturing presents restrictions in lead times, process outsourcing, or associated costs.

The use of anatomical models for preoperative planning is an interesting utility in all surgical specialties [49]. Anatomical models are replicas of a patient-specific organ or anatomical region manufactured in different materials with millimetric accuracy. They are used as a communication tool with patients or between professionals [3], facilitate decision-making, and allow simulation before the surgical procedure [2]. These simulators of medical techniques such as orotracheal intubations [52], sutures [53], endoscopies [54], endovascular interventions [55], or other surgical procedures [56–58] can be developed at a very low cost. They can also be an alternative to corpses for teaching anatomy [59–61].

**Fig. 10:**
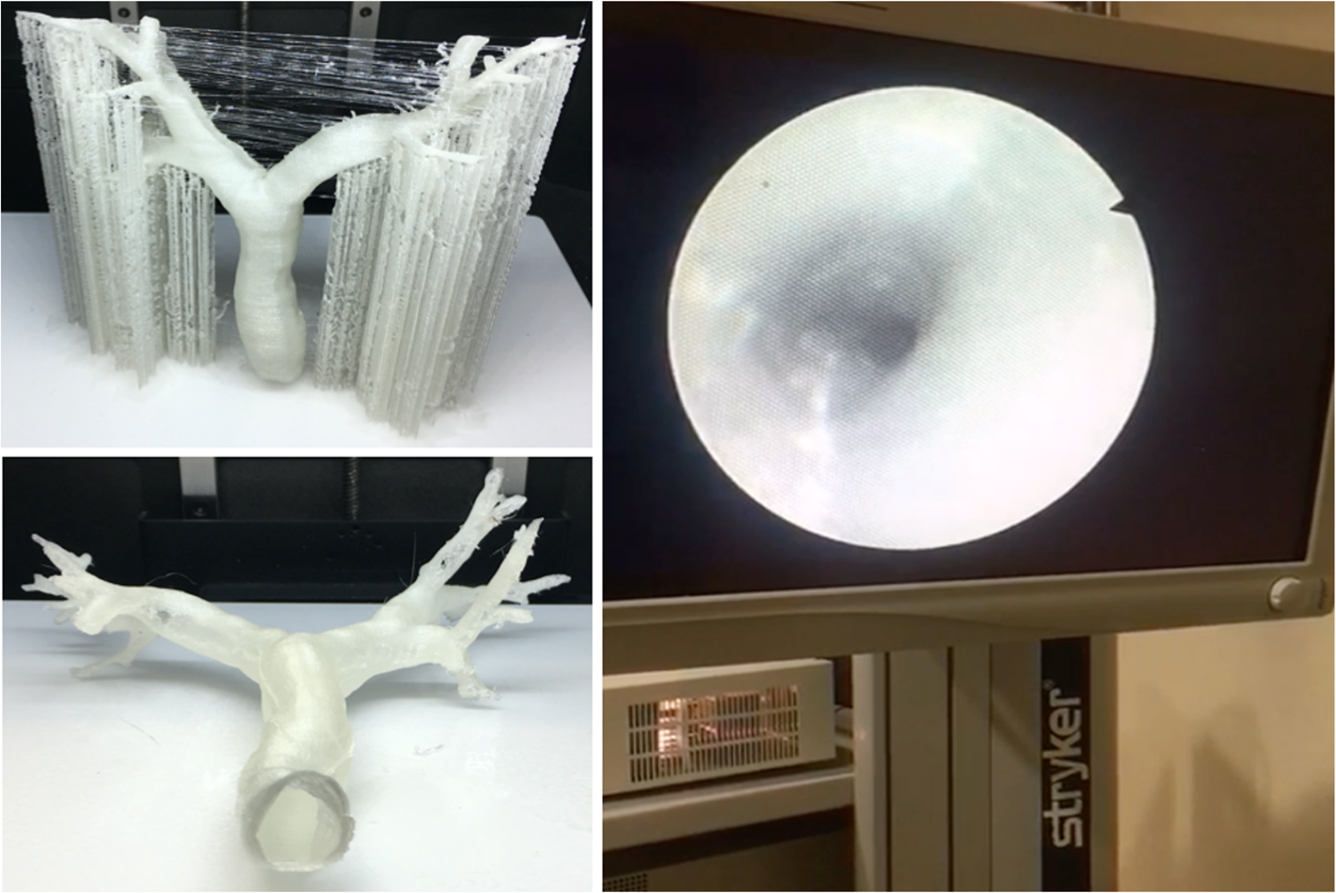
Airway simulator. Date, 2017

**Fig. 11:**
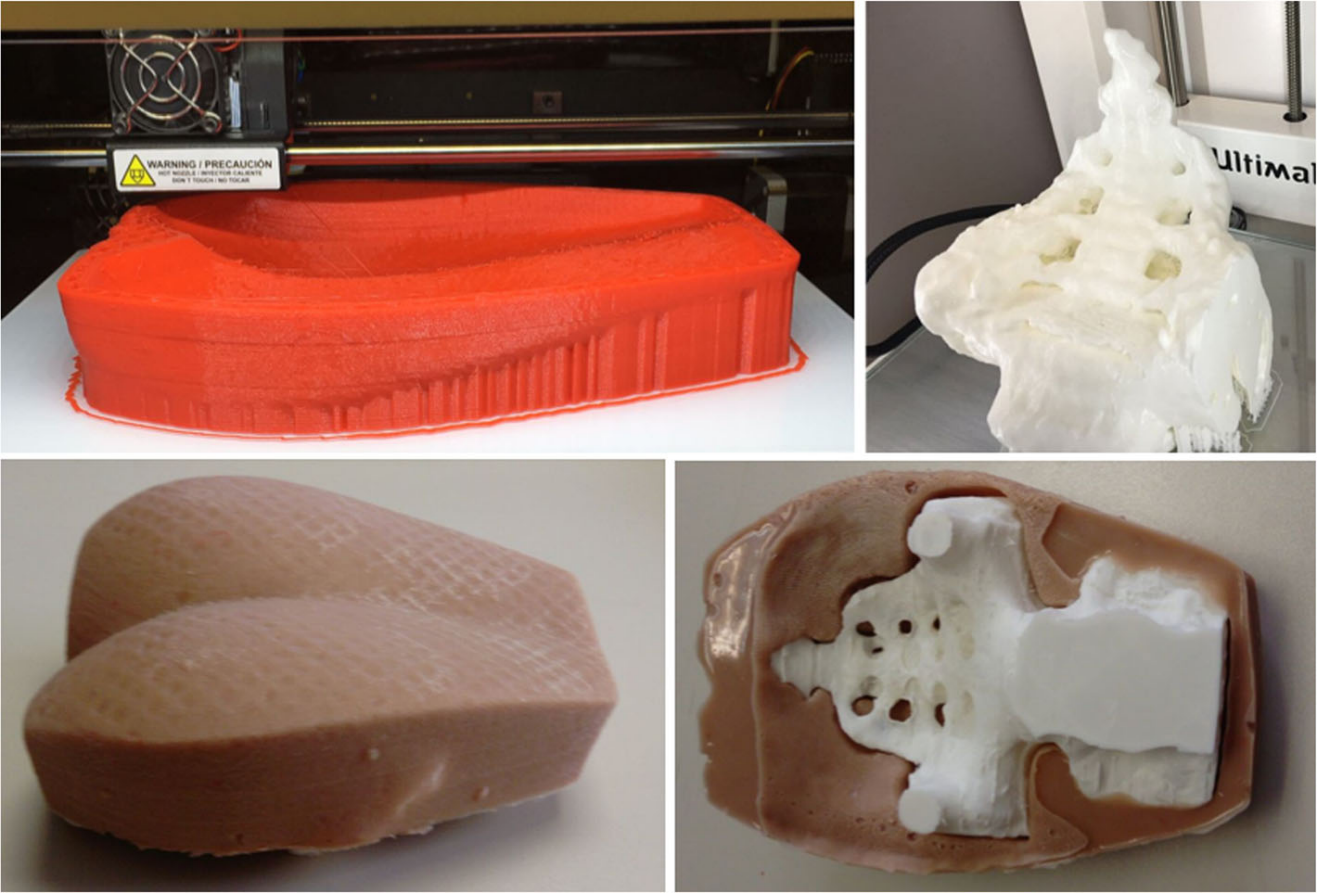
Simulation of interventional procedures. Sacral root neuro stimulation. Date, 2016

**Fig. 12:**
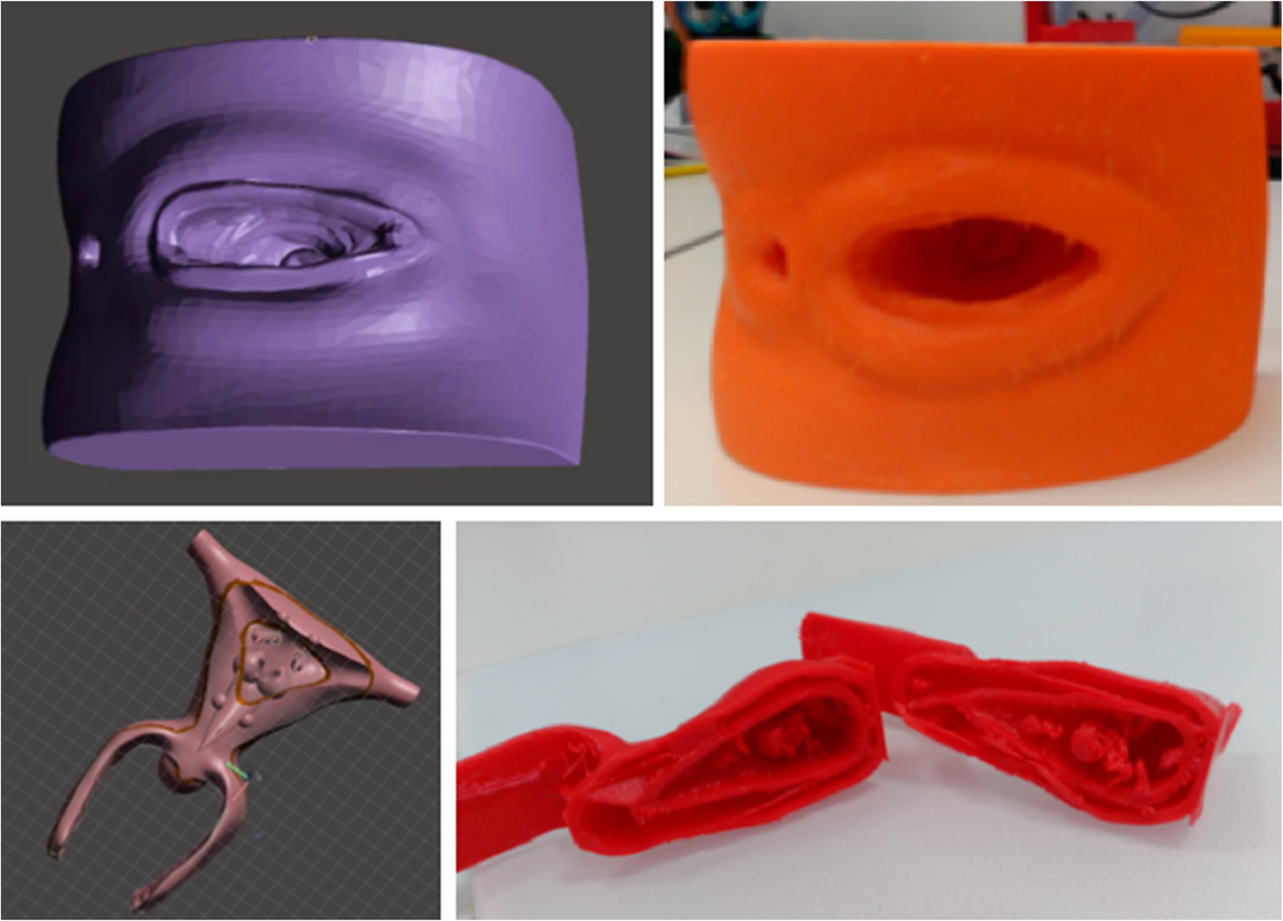
Simulators for gynecological training. Date, 2016

**Fig. 13:**
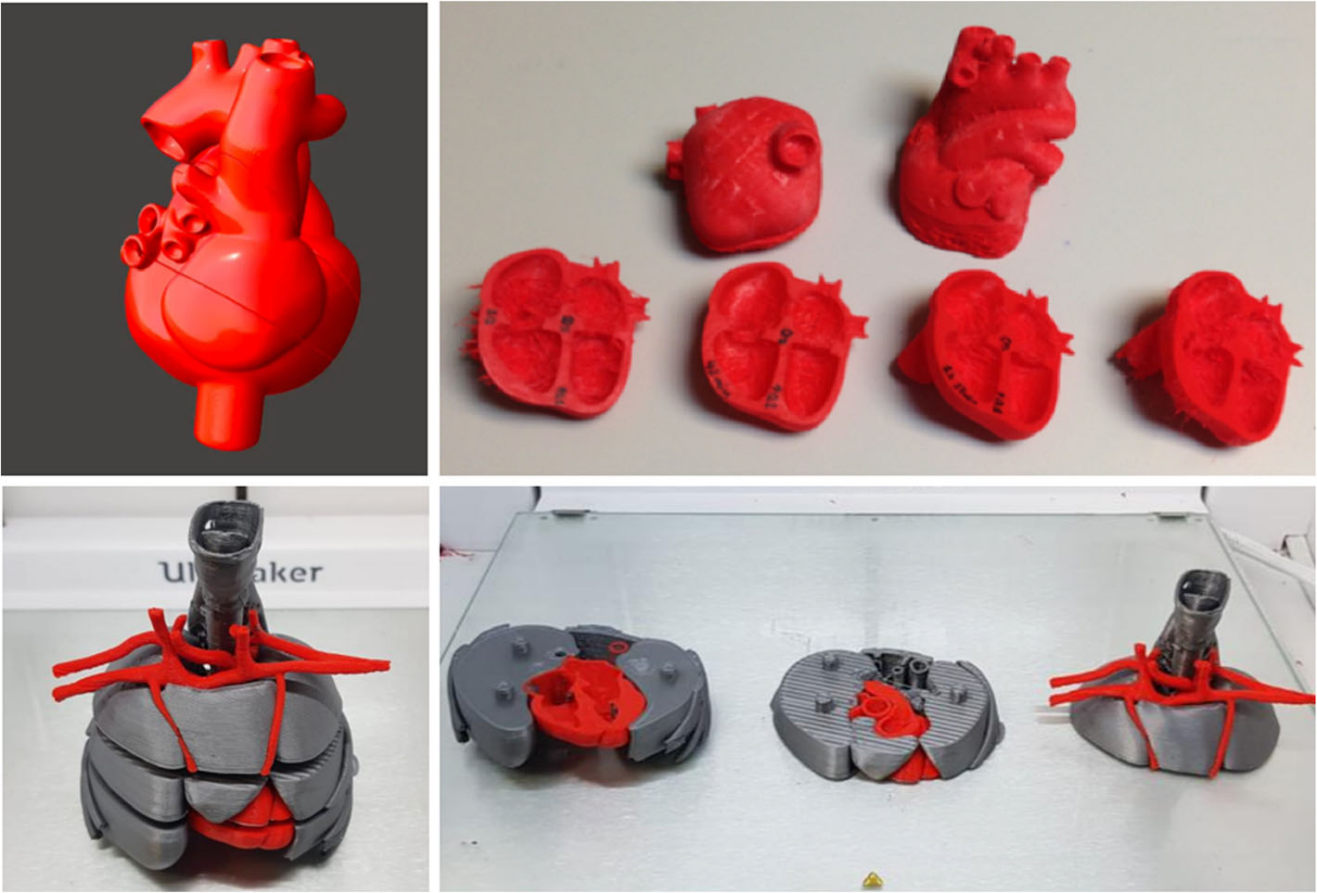
Anatomical models for teaching. Fetal cardiology. Date, 2019

**Fig. 14:**
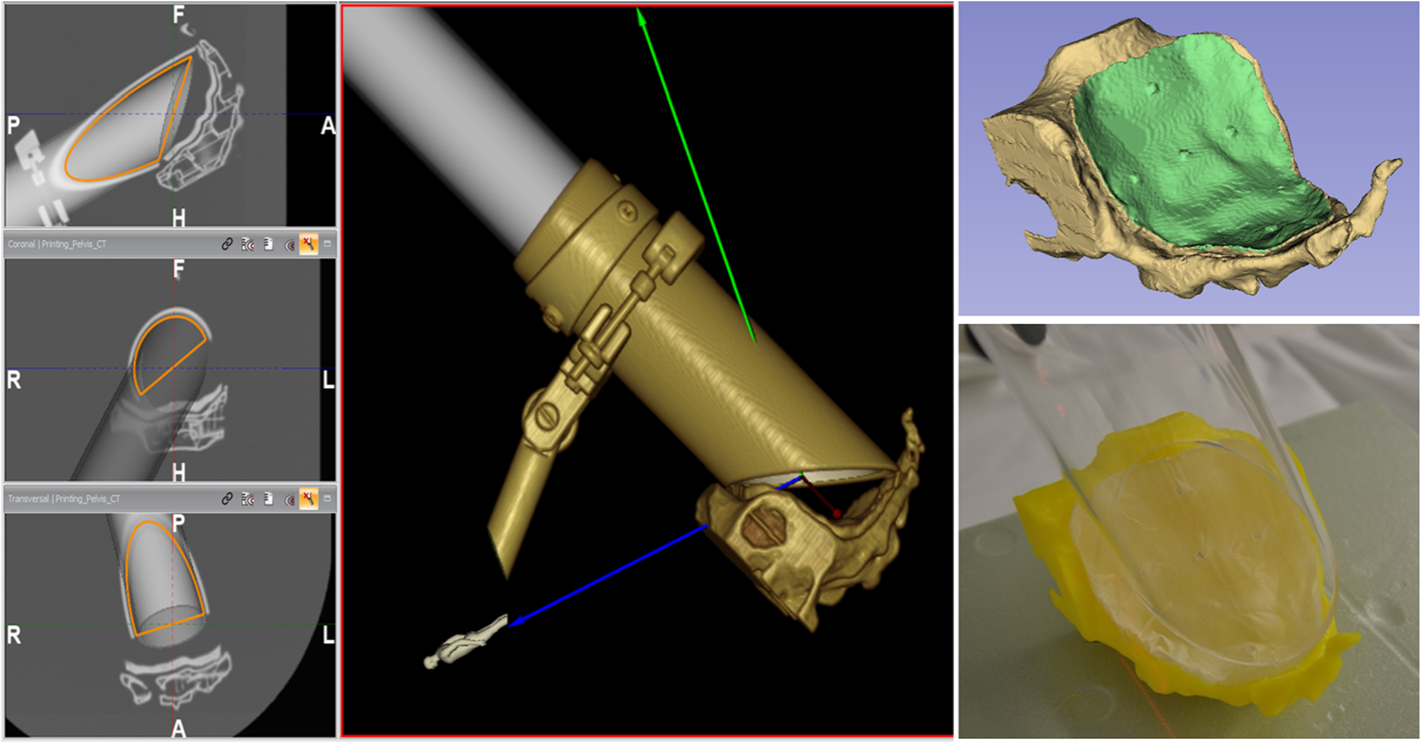
Sacral tumor. Radiotherapy treatment simulation. Dose measurement. Date, 2017

In this study, 86.80% of the required products were anatomical models, useful not only in surgical planning but also as a communication or research tool. In the same way that a university hospital complements university education enriching the academic environment, a manufacturing university hospital does not replace factories. In this setting, 3D printing goes hand-in-hand with translational research and teaching, acting as an accelerator for clinical innovation. Figures 10, 11, 12, and 13 show examples of this approach for medical training. Our “in-house” 3D printing approach has allowed complete control and monitoring of the process, from the indication to the manufacture of a customized medical-surgical solution. It also presents added value when manufacturing guides, instruments, or customized implants [62–64]. As an example, Fig. 14 shows a complex co-design with radiation oncologists and physicists to evaluate intraoperative radiation therapy dose on patient-specific data.

**Fig. 15:**
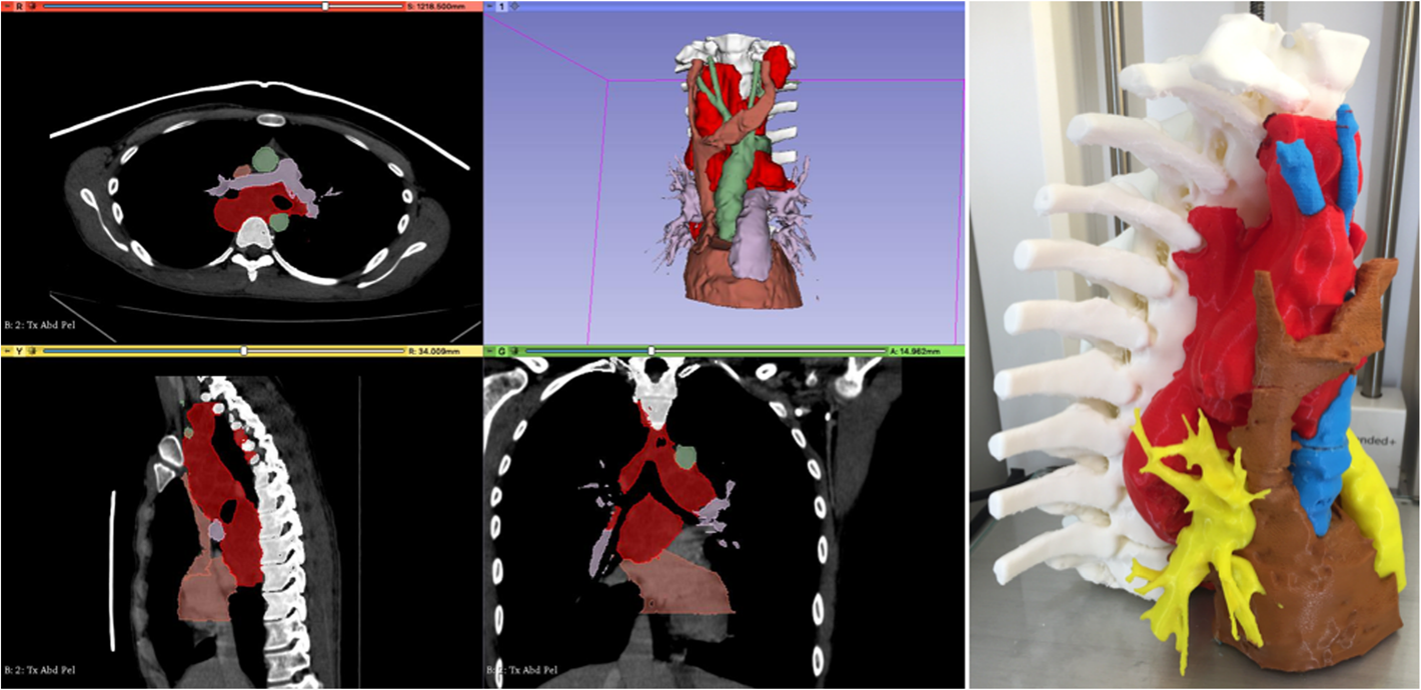
Anatomical model of a complex anatomical area. Date, 2019

The technical details in Table 3 show how the duration of the process depended on the type of product required and the input data received. It may seem that work time should be lower for an anatomical model than for a surgical guide, since the last requires a specific design. However, the average work time showed no significant differences between these two products in our study. This can be explained by the segmentation and image processing time required by anatomical models, which can be quite laborious for complex anatomical areas, as shown in Fig. 15. Anatomical models showed both long printing times and a large amount of material compared to surgical guides. We also observed an increase in 3D printing time in the last 2 years, justified by the complexity of 3D-printed products, which usually required the optimization of manufacturing parameters with different materials.

The availability of machines for “in-house” manufacturing by material extrusion and vat photopolymerization has allowed the production of anatomical models, surgical guides, and patient-specific instruments. A multidisciplinary team, with valuable experience and clinical knowledge, achieved low response times, maintaining the enrollment of most hospital departments in the POC manufacturing model. This approach is aligned with the current regulatory framework of this technology applied to personalized medicine, which identifies the prescribing physician as the final responsible for the process [65–68].

## Conclusions

This study identifies the possibilities of integrating 3D printing technology in a hospital. This experience shows the conceptual evolution of the hospital’s 3D printing workflow toward POC manufacturing. Our results are limited to a single center, so it is essential to propose multicenter studies and elaborate consensus documents to take advantage of the upcoming contributions of 3D printing in healthcare.

## Data Availability

The authors declare that they have followed their center’s protocols on the publication of patient data. All data analyzed during the current study are available from the corresponding author on reasonable request.

## Abbreviations

3D: Three dimensional
3DP: Three-dimensional printing
ABS: Acrylonitrile butadiene styrene
COVID-19: Coronavirus disease 2019
DICOM: Digital imaging and communication on medicine
FDM: Fused deposition modeling
GIRFT: Getting It Right First Time
ISO: International Organization for Standardization
PLA: Polylactic acid
POC: Point of care
POCM: Point-of-care manufacturing
PVA: Polyvinyl alcohol
SLA: Stereolithography
TPU: Thermoplastic Polyurethane
UPAM3D: Advanced Planning and 3D Manufacturing Unit
